# The Prevalence of Insulin Resistance and the Associated Risk Factors in a Sample of 14–18-Year-Old Slovak Adolescents

**DOI:** 10.3390/ijerph18030909

**Published:** 2021-01-21

**Authors:** Jana Jurkovičová, Katarína Hirošová, Diana Vondrová, Martin Samohýl, Zuzana Štefániková, Alexandra Filová, Ivana Kachútová, Jana Babjaková, Ľubica Argalášová

**Affiliations:** Institute of Hygiene, Faculty of Medicine, Comenius University in Bratislava, Spitalska 24, 813 72 Bratislava, Slovakia; jana.jurkovicova@fmed.uniba.sk (J.J.); katarina.hirosova@fmed.uniba.sk (K.H.); diana.vondrova@fmed.uniba.sk (D.V.); martin.samohyl@fmed.uniba.sk (M.S.); zuzana.stefanikova@fmed.uniba.sk (Z.Š.); alexandra.filova@fmed.uniba.sk (A.F.); ivana.kachutova@fmed.uniba.sk (I.K.); jana.babjakova@fmed.uniba.sk (J.B.)

**Keywords:** adolescents, cardiometabolic risk factors, insulin resistance, abdominal obesity, lifestyle, dietary habits

## Abstract

The prevalence of cardiometabolic risk factors has increased in Slovakian adolescents as a result of serious lifestyle changes. This cross-sectional study aimed to assess the prevalence of insulin resistance (IR) and the associations with cardiometabolic and selected lifestyle risk factors in a sample of Slovak adolescents. In total, 2629 adolescents (45.8% males) aged between 14 and 18 years were examined in the study. Anthropometric parameters, blood pressure (BP), and resting heart rate were measured; fasting venous blood samples were analyzed; and homeostasis model assessment (HOMA)-insulin resistance (IR) was calculated. For statistical data processing, the methods of descriptive and analytical statistics for normal and skewed distribution of variables were used. The mean HOMA-IR was 2.45 ± 1.91, without a significant sex differences. IR (cut-off point for HOMA-IR = 3.16) was detected in 18.6% of adolescents (19.8% males, 17.6% females). IR was strongly associated with overweight/obesity (especially central) and with almost all monitored cardiometabolic factors, except for total cholesterol (TC) and systolic BP in females. The multivariate model selected variables such as low level of physical fitness, insufficient physical activity, breakfast skipping, a small number of daily meals, frequent consumption of sweetened beverages, and low educational level of fathers as significant risk factors of IR in adolescents. Recognizing the main lifestyle risk factors and early IR identification is important in terms of the performance of preventive strategies. Weight reduction, regular physical activity, and healthy eating habits can improve insulin sensitivity and decrease the incidence of metabolic syndrome, type 2 diabetes, and cardiovascular disease (CVD).

## 1. Introduction

Cardiovascular diseases (CVDs) are one of the most serious problems related not only to health but also to the social and economic situation in all developed countries of the contemporary world [1], and this unfavorable epidemiological situation is significant in Slovakia as well. CVDs are still the most common chronic diseases and, in the long run, they hold a leading position in the causes of death (nearly 50%) as well as in the causes of hospitalization in the Slovak population [2].

CVDs are often considered to be diseases of the elderly, but many epidemiological studies have shown early stages of the atherosclerotic process in childhood and adolescence [3]. At this age, individuals with atherosclerotic lesions cannot be identified by clinical signs only, so attention should be focused on biochemical markers (particularly plasma lipid levels), anthropometric examinations, and blood pressure level measurements, which help to reveal risk indicators of atherogenesis [4]. The youngest age groups should become the target groups of interventions aimed at reducing cardiovascular risk, because childhood and adolescence are extremely sensitive periods of life in terms of the effects of cardiometabolic risk and the development of atherosclerosis, hypertension, and diabetes in adulthood [5].

Insulin resistance (IR) can be defined as a condition in which peripheral tissues do not respond sufficiently to insulin, which gradually leads to impaired glucose tolerance and later to the development of type 2 diabetes. Insulin resistance can also be considered as a separate risk factor for CVD and is associated with other metabolic diseases. Insulin resistance is a central risk factor for metabolic syndrome (MetS) and stimulates the development of other cardiometabolic risk factors such as dyslipidemia, arterial hypertension, hyperglycemia, and other pathological conditions of the organism. One of the first IR warning signals is the proliferation of adipose tissue as an important endocrine organ, especially in the waist area [6]. Thus, IR is closely linked to abdominal obesity. In addition to abdominal obesity, high levels of fasting insulin are present, by which the organism tries to overcome the biological effect of insulin reduction [7,8].

Metabolic syndrome is a phenomenon of modern society [9]. It is commonly defined as a grouping of chronic diseases that include obesity, type 2 diabetes, hypertension, dyslipoproteinemia, and CVD [10]. It develops in individuals with a genetic predisposition and with an inappropriate lifestyle, i.e., in the case of excessive energy intake, insufficient physical activity, etc. Various cardiovascular and metabolic risk factors for atherosclerosis, and its complications, arise gradually.

Although MetS was originally described in adults and was not known in children before 1980, the clustering of cardiometabolic risk factors begins at a young age and, currently, the same metabolic diseases found in adults can be found in 13% of normal-weight children and 38% of children with obesity [5,10]. Early identification of risk factors is very important to allow preventive actions. However, the selection and definition of these risk factors with cut-off values are not without discussion and controversy, especially in the pediatric population, where cut-off values for homeostasis model assessment (HOMA-IR) are very variable and the definition of MetS is even more complicated [11]. These complications arise not only because children are in the early stages of cardiovascular and metabolic changes, but also because of physical development and adolescence. Therefore, age-corrected cut-off values should be considered [10].

MetS prevalence in children is lower than in adults, estimated at 2–9.4%, but it is higher in children and adolescents with obesity (12.4–44.2%) [12]. Existing definitions of MetS for children have been derived from definitions for adulthood on the assumption that conditions are similar throughout life [13,14].

In 2007, the International Diabetes Federation (IDF) presented the definition of MetS in children and adolescents [15]. It provides a standard that simplifies the comparison of the results of individual studies because the use of different definitions of MetS in children and adolescents has, in the past, led to significant differences in MetS prevalence in the international literature from 0% to 59% [16].

In the last few decades, many interventional studies have gained a wealth of new knowledge about foods and nutrients that positively or adversely affect the cardiovascular system not only in adults [17] but also in adolescents [18]. Healthy nutrition plays an important role in both primary and secondary CVD prevention and has a beneficial effect on plasma lipid levels [17]. Adverse associations of cardiovascular health with increased intake of sodium, saturated fats, meat, fast foods, and sweetened beverages have been reported in children and adolescents [19]. Children with a higher intake of sweetened beverages had a 55% higher risk of overweight/obesity compared to children with a lower intake. One to two servings of sweetened beverages per day were associated with an increased risk of type 2 diabetes development by 26% compared to occasional consumption (less than one serving per month) [20]. Even moderate sweetened beverages consumption (>1.3 cups per day) can be an important cardiometabolic risk predictor of young people, regardless of their weight [21]. 

Physical inactivity doubtless belongs to CVD risk factors. Insufficient physical activity is also significantly associated with the development of MetS and is probably a decisive factor in IR development [22]. Increasing physical activity in all age categories is an important factor in preventive cardiology [23] and can effectively improve cardiovascular morbidity and mortality. Regular physical activity in childhood and adolescence is especially important because of the formation of lifelong habits [24]. Low aerobic fitness in children and adolescents is associated with a higher prevalence of cardiometabolic risk factors (abdominal obesity, elevated blood pressure, increased levels of insulin, glucose and plasma lipids, increased IR prevalence, and enlarged left ventricle of the heart) [24,25,26,27].

Sleep has an essential role in growth, maturation, and good health during childhood and adolescence [28]. However, shortening and reduced sleep quality is a common problem in children and adolescents [29]. Examination of more than 4000 adolescents has shown that, in healthy adolescents, sleep disorders are associated with abnormalities in CVD risk factors—elevated total cholesterol (TC) levels, higher body mass index (BMI), and hypertension [30,31]. The results of a study of almost 7000 Lithuanian children aged 12–15 years showed that short sleep (<7 h) was significantly associated with an increased risk of prehypertension and hypertension [29]. An analysis of 25 studies showed that all studies indicated significant associations between sleep duration and childhood overweight and obesity [32]. Intervention strategies aimed at optimizing sleep hygiene in childhood and adolescence may therefore play an important role in terms of CVD prevention in adulthood.

This cross-sectional study aimed at determining the prevalence of IR in adolescents and its relationship with obesity and other cardiometabolic and selected lifestyle risk factors, as well as selected factors of personal and family history in males and females. These indicators are of great importance for CVD prevention in adolescents and young adults.

## 2. Materials and Methods 

### 2.1. Study Design 

The analyzed sample of adolescents was selected from the project “The Support of Cardiometabolic Health in the Secondary Schools in the Bratislava Self-governing Region” (Respect for Health), which was conducted at secondary schools within the Bratislava Self-governing Region (14 grammar schools and 48 secondary vocational schools). The project was approved by the Ethics Commission of the Bratislava Self-Governing Region. All procedures were according to the ethical standards, as laid down in the 1964 Declaration of Helsinki and its later amendments. From all legal representatives of children, written informed consent was obtained before being enrolled in the study. Participation in the study was fully voluntary and anonymous, with no explicit incentives provided for participation. All participants were informed about the significance and the course of individual measurement and examination procedures.

From all of the students enrolled in high schools in the Bratislava Self-Governing Region (n = 19,172; 9821 males, 9351 females), 4382 adolescents (2097 males, 2285 females) aged 11–19 years participated in this project (22.9%). Students < 14 and >18 years were excluded. Selection criteria were age (decimal age 14.00–18.99 years according to WHO criteria) and complete anthropometric, blood sample, and blood pressure (BP) examinations. An exclusion criterion was the presence of any illness (acute and/or chronic). The final selected and evaluated sample consisted of 2629 adolescents aged 14–18 years with a mean age of 17.1 ± 1.04 years: 1205 boys (45.8%) and 1424 girls (54.2%). The basic characteristics of the adolescents and sex differences are shown in Table 1.

### 2.2. Anthropometric Measurements

Basic anthropometric measurements, focused on overweight/obesity and abdominal obesity (i.e., weight, height, waist circumference, body fat content), were performed by trained staff directly at schools. Body fat content and weight were measured using a digital body composition scale (OMRON BF510) with an accuracy of 100 g. The subject stood in light clothing on metal electrodes with bare feet, their palms firmly gripping handles with electrodes, and their hands were freely lowered (we subtracted 500 g for clothing from the measured value). The body fat percentage measurement was based on the bioelectrical impedance method. The body height was measured using a stadiometer (TANITA LEICESTER) on a horizontal floor surface with an accuracy of a whole millimeter (the measured person was barefoot, upright, their feet and shoulders touched the scale, and their head was upright). Body mass index was calculated; overweight and obesity was evaluated according to the percentile curves from the 6th National Anthropometric Survey on physical development of children and youth in the Slovak Republic in the year 2001 [33]. Subjects below the 3rd percentile were classified as underweight, subjects above the 90th percentile as overweight, and those above the 97th percentile were classified as obese. Body fat content (category underweight, normal weight, overweight, obesity) was evaluated according to McCarty [34].

Waist circumference was measured using a flexible non-elastic tape in an expiration position directly on the skin in the horizontal plane at half the distance between the *crista iliaca anterior superior* and the lowest rib (with accuracy to the whole millimeter) and evaluated according to the IDF consensus for defining MetS in children and adolescents [15]. From the measured data, waist-to-height ratio (WHtR) was calculated; WHtR > 0.5 was evaluated as central obesity. 

### 2.3. Blood Pressure and Resting Heart Rate Measurement

Blood pressure and resting heart rate (HR) were measured using an automated digital device, OMRON M-6 COMFORT, under standard conditions on the right arm (after a minimum of 10 min of sitting with back supported and legs on the floor). Before the examination, subjects did not eat or smoke for 2 h. The average values of BP and HR from the 2nd and the 3rd measurements (with a 5-min break between measurements) were calculated. BP values at the age of <18 years were classified using percentile values of systolic and diastolic BP according to age, height, and sex [35,36] and, in 18-year-olds, according to the adult population classification [37].

### 2.4. Biochemical Blood Analyses

A sampling of overnight fasting venous blood for laboratory examination was performed by qualified nurses under the physician’s guidance and standard biochemical analyses were performed by a central certified laboratory. At the central laboratory, standard blood chemistry analyses (plasma fasting glucose, insulin level, total cholesterol, triglycerides (TG), high-density lipoprotein cholesterol (HDL-C) plasma levels, concentrations of high-sensitive C-reactive protein (hsCRP)) were performed (ADVIA 2400 analysers, Siemens, Erlangen, Germany). Low-density lipoprotein cholesterol (LDL-C) level was calculated via the Friedewald equation; atherogenic index of plasma (AIP) = log(TG/HDL-C) [38] and (HOMA-IR) [39,40] were calculated as well. 

Plasma lipids levels were evaluated according to the recommendations for the diagnosis and treatment of dyslipidemias in children and adolescents in the Slovak Republic [3]. Non-HDL-C levels were classified according to cut off points of the Expert Panel on Integrated Guidelines for Cardiovascular Health and Risk Reduction in Children and Adolescents [41]. AIP > 0.21, glycaemia ≥ 5.6 mmol/L, hsCRP ≥ 3 mg/L, and insulin > 20.0 mIU/L were considered as high risk levels. Cut-off point ≥ 3.16 for HOMA-IR was considered to be risky for insulin resistance [39]. 

The MetS prevalence was evaluated according to the IDF consensus for the definition of MetS in children and adolescents, separately in the age group < 16 years and 16 + years [15] as the presence of central obesity plus two or more other components (elevated BP, TG, glycemia, and/or reduced HDL-C level).

### 2.5. Physical Fitness Assessment

The overall physical fitness was assessed using the Ruffier test, which is used to assess cardiovascular fitness [42]. Fitness was evaluated as follows: Ruffier index (RI) = 0—excellent, 0.1–5—good, 5.1–10—average, 10.1–15—weak, and >15—insufficient.

### 2.6. Questionnaires

The objective examination was supplemented by a comprehensive student’s questionnaire, where we focused mainly on selected lifestyle characteristics: smoking (current = any in the past month/former), leisure-time physical activity (frequency and duration per week), sleeping duration and sedentary activities duration (working/gaming at the computer, watching TV, learning) on working days and weekends, psychosocial factors (stress situations experiencing at school and in privacy: frequently—occasionally, exceptionally, or no), and nutrition and dietary habits: number and regularity of daily meals (regularly/daily, irregularly/occasionally, or never), frequency of food groups’ consumption per week (daily, 3–6-times, 1–2-times, or rarely/never). In this study, we recorded the consumption of only sweetened beverages. In the questionnaire designed for parents, we focused on the adolescent birth weight and the breastfeeding duration, the current weight of the parents, and their highest educational level. Multiple response options were dichotomized (desirable/acceptable vs. risky) for multivariate logistic regression. 

### 2.7. Statistical Analyses

For statistical data processing, the methods of descriptive statistics for normal (frequencies, means ± standard deviations) and skewed distribution of variables (median, interquartile range) were used. Relationships between continuous variables were assessed using a two-sample *t*-test and Mann–Whitney U-test, categorical data were compared using contingency tables and chi-square test. The relationship between IR and each of all of the cardiometabolic factors (total cholesterol, triglycerides, AIP, HDL-C, non-HDL-C, LDL-C, hsCRP, systolic/diastolic BP, body fat content, WHtR), lifestyle factors (smoking, physical activity, sleeping duration, sedentary activities duration, stress, meals and breakfast consumption frequency), and selected data on personal and family history (sex, age, breastfeeding duration, education and current weight of parents) was tested using bivariate analysis, which resulted in crude odds ratios at the 95% confidence interval.

Multivariate logistic regression models were used as well (separately for the cardiometabolic, lifestyle, and personal/family history variables), which resulted in adjusted odds ratios at the 95% confidence interval; *p*-values < 0.05 were considered as statistically significant. In the case of multivariate logistic regression, collinear variables were excluded (LDL-cholesterol, diastolic BP, BMI, and waist circumference). All variables were entered into a general logistic backward stepwise regression model and the elimination method was used to remove non-significant predictors from the model in 16 steps. The stepwise approach is useful because it reduces the number of predictors, reducing the multicollinearity problem, and it is one of the ways to resolve overfitting. The statistical packages Epi InfoTM software, version 7.1.5.0, Atlanta, GA, USA, and SPSS, version 24 (International Business Machines Corp., New Orchard Road, Armonk, NY, USA) were applied.

## 3. Results

The basic sample characteristics, sex differences, and risk marker prevalence are shown in Table 1 (the average age of boys and girls was the same, i.e., 17 years). The table shows significant sex differences for almost all monitored parameters, except for the level of hsCRP and the HOMA-IR (no significant differences between sexes). Males displayed significantly higher values of anthropometric measurements, except for the total body fat percentage, higher values of the atherogenic index, glycemia, and systolic/diastolic BP. Significantly higher body fat content, higher levels of TC, LDL-C, HDL-C, non-HDL-C, TG, plasma insulin, and HR in females were presented.

According to BMI, males had a significantly higher prevalence of overweight/obesity (30.7% vs. 22.9%; *p* < 0.001) but, according to body fat content, females had a significantly higher prevalence of overweight (14.0% vs. 7.9%, *p* < 0.001). The abdominal obesity according to the IDF criteria [15] was more frequent in females (not significant) but, according to WHtR, was significantly more frequent in males (12.3% vs. 8.4%, *p* < 0.001).

The Ruffier fitness test showed significant sex differences and overall poor fitness of adolescents, which was significantly worse in girls. The average RI for boys was 9.2 ± 4.0 (in the range of average fitness), and for girls it was 10.5 ± 4.1 (in the range of weak fitness), *p* < 0.001.

Selected lifestyle and eating habit characteristics are given in Table 2. Females reported a significantly higher prevalence of smoking, shorter leisure-time physical activity duration per week, and, conversely, longer durations of sedentary activities (working/gaming at the computer, watching TV, learning), more frequent stressful situations (at school, in privacy), a lower average number of meals per day, and more frequent breakfast skipping.

**Figure 1:**
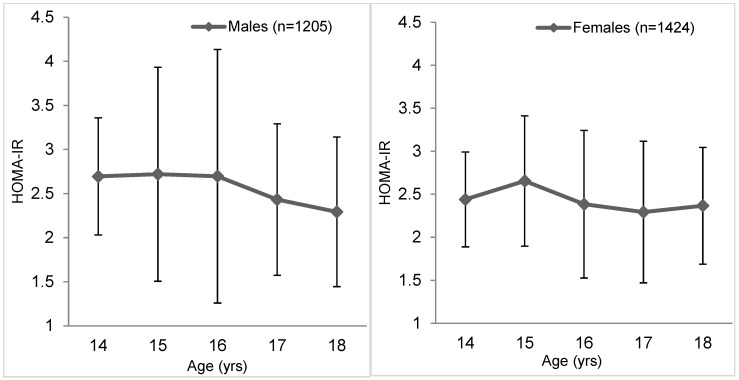
The mean HOMA-IR values in adolescents according to age categories (means and standard deviations).

**Figure 2:**
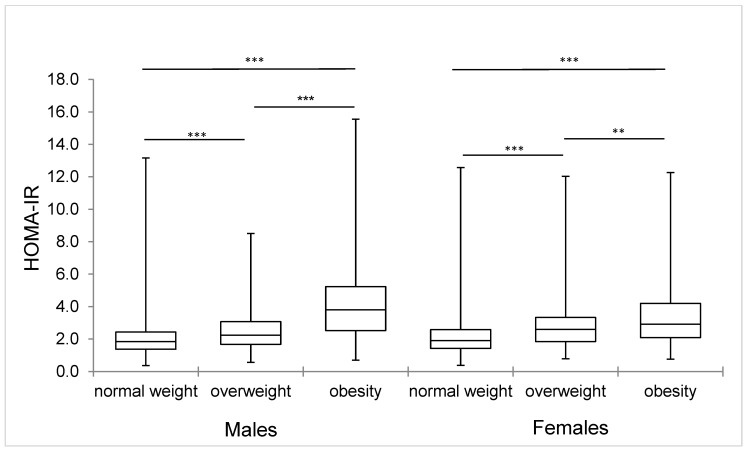
HOMA-IR values box-plot representation considering median values and 25–75 percentile distributions according to BMI categories and the pair-wise comparison. *** *p* = 0.000; ** *p* = 0.004.

The mean HOMA-IR in the whole cohort was 2.45 ± 1.91 (males 2.53 ± 2.25 and females 2.39 ± 1.58). Mean HOMA-IR values were higher in 14- to 17-year-old males and in 18-year-old females and had a declining trend in both sexes (Figure 1). Insulin resistance according to the HOMA-IR was observed in a total of 18.6% of adolescents (19.8% of boys and 17.6% of girls, *p* = 0.147), the median of HOMA-IR increases significantly regarding the overweight and obesity prevalence (Figure 2). The IR prevalence in adolescents with normal weight was 11.6% and for overweight it was 26.3%, but in obese it was up to 56.3%.

In the whole sample (n = 2629), 38 (1.4%) adolescents met the IDF criteria for the MetS diagnosis (32 boys, 2.7%, and 6 girls, 0.4%, *p* < 0.001). All of them were in the range of overweight/obesity. In the overweight/obesity group (n = 696), the MetS prevalence was 5.5% (8.6% of boys and 1.8% of girls, *p* < 0.001).

Bivariate analysis showed highly significant associations of IR with each of all observed cardiometabolic factors in boys. This was similar in the case of girls, except for TC level and systolic BP. Among the metabolic parameters, in both sexes, the closest relationships of HOMA-IR with the TG level (OR = 5.27; 95% CI 3.79, 7.32; *p* = 0.000 in males; OR = 2.28; 95% CI 1.67, 3.09; *p* = 0.000 in females) and AIP (OR = 5.51; 95% CI 3.64, 8.34; *p* = 0.000 in males; OR = 3.65; 95% CI 2.20, 6.06; *p* = 0.000 in females) were observed, and with all anthropometric parameters, especially waist circumference (OR = 13.48; 95% CI 8.76, 20.76; *p* = 0.000 in males; OR = 3.70; 95% CI 2.64, 5.19; *p* = 0.000 in females) and WHtR (OR = 9.88; 95% CI 6.80, 14.37; *p* = 0.000 in males; OR = 3.98; 95% CI 2.68, 5.90; *p* = 0.000 in females). 

Using the information provided by the bivariate analysis, multivariate analyses were performed to adjust the model for some confounding variables and collinear variables were excluded. Triglyceride levels (AOR = 3.77; 95% CI = 2.28, 6.22; *p* = 0.000), body fat content (AOR = 2.64; 95% CI = 1.66, 4.21; *p* = 0.000), and WHtR (AOR = 3.25; 95% CI = 1.91, 5.51; *p* = 0.000) in males and non-HDL-cholesterol (AOR = 1.51; 95% CI = 1.001,2.26; *p* = 0.049), triglycerides (AOR = 1.60; 95% CI = 1.08, 2.38; *p* = 0.020), body fat content (AOR = 2.29; 95% CI = 1.65, 3.19; *p* = 0.000), and WHtR (AOR = 1.96; 95% CI = 1.24, 3.11; *p* = 0.004) in females were statistically significant among the variables associated with IR in adolescents in multivariate logistic regression analyses (Table 3).

Regarding lifestyle factors, multivariate analysis revealed significant associations of IR with insufficient physical fitness and inadequate physical activity in both sexes (Table 4). In addition, skipping breakfast and insufficient sleep time during weekends in males, a low number of meals a day, and a frequent consumption of sweetened beverages in females showed significant associations with IR. Paradoxically, we found significant inverse associations of IR with smoking in boys and with frequent stressful situations at school in girls.

In terms of personal and family history factors, low birth weight showed marginally significant association with IR only in bivariate analysis and only in males (OR = 1.87; 95% CI = 0.93, 3.75; *p* = 0.07). Multivariate logistic regression confirmed a significant association of IR with a higher educational level of mothers and with a higher current weight of both parents in males, and with a lower educational level of fathers in females (Table 5). 

Finally, a backward age and sex-adjusted elimination logistic regression model was performed; non-significant variables were removed stepwise from the model (Table 6). This model showed a significant association of IR with increased TG level, body fat content, and WHtR among cardiometabolic risk factors (association with lower HDL-C level revealed only borderline significance) with poor physical fitness, insufficient physical activity duration per week, non-smoking status, the infrequent occurrence of stressful situations at school, irregular/no breakfast consumption, and frequent sweetened beverages consumption among lifestyle risk factors, and with a lower educational level of fathers and a higher educational level of mothers among family factors.

## 4. Discussion

The CVD prevalence in Slovakia is shifting to younger age groups. Risk factors accelerating the development of these diseases have been active since early childhood. Most of the main risk factors playing a key role in CVD development are significantly related mainly to lifestyle factors such as smoking, unhealthy nutrition, and physical inactivity [35]. 

The most common causes of IR are overweight/obesity, lack of physical activity, poor diet, excessive fructose intake, and smoking [43]. Insulin resistance is a major pathogenetic mechanism associated with a predisposition to premature CVD. Several indices are used to assess IR, e.g., HOMA, or to assess insulin sensitivity, e.g., Quantitative Insulin Sensitivity Check Index (QUICKI). In our study, we used the HOMA-IR, given that this index is more reliable and credible in children and adolescents and has been used as a measure of IR in this population group [39]. This index was first introduced by Matthews et al. [44] in 1985, and its use is advantageous because it is a practical, fast, and inexpensive method. We considered a cut-off point of ≥3.16 [39] to be risky in terms of IR. In adults, HOMA-IR values ≥ 2.5 are considered in IR, but the exact cut-off value for children and adolescents has not been established [45]. In several papers, the cut-off values for adolescents range from about 2.2 to 5.3, but these studies vary considerably in design, sample size, age, nutritional status, and the stage of puberty of their participants [45]. In a 2015 survey [46], the lowest cut-off value for HOMA was found to be 1.65 for girls and 1.95 for boys [47], and the highest was 3.82 for girls and 5.22 for boys [48]. Van der Aa et al. [49] even report limit values of 1.14–5.56, resulting in a wide range of IR prevalence, from 5.5% to 72.3%. This wide variation of cut-off values also makes it very difficult to compare the prevalence of IR in different countries and ethnic groups, and it is not entirely clear which cut-off value is best for defining IR in adolescents.

In our sample, almost one fifth of adolescents, 18.6% (19.8% of boys and 17.6% of girls), had a risk of IR without significant sex differences in average HOMA-IR values (2.53 boys, 2.39 girls, *p* = 0.077). The same cut-off value was used by authors in Košice (Slovakia) in a group of 224 high school students with an average age of 18 years, and they found only a 13.9% prevalence of IR [50]. The IR prevalence in a relatively small sample of Brazilian children (n = 162) aged 12–18 years was slightly higher, 23.5% at the same cut-off point of 3.16, but at overweight/obesity there was a prevalence of 45.7% [51]. However, if we used the lowest recommended cut-off values [47], the prevalence of IR would increase to 53.7% (males) and 68.3% (females). This is questionable for the overweight/obesity prevalence in our cohort (30.7% males/22.9% females). Slightly higher average values of HOMA-IR (2.77 in boys and 2.93 in girls) were found by the authors in a group of 691 healthy adolescents from urban areas in India [52], although it was a slightly younger age group (10- to 17-year-olds). The shape of the curve of HOMA-IR average values in different age groups was also different. In Indian adolescents, the lowest values were found at the age of 13 with a subsequent increase and maximum values at the age of 17. In our sample, the course was opposite; after the age of 15, the average HOMA-IR values decreased with the lowest values at the age of 17 years (in females) and 18 years (in males).

Adolescence is a critical period for the onset of obesity and other metabolic disorders associated with body fat accumulation. Overweight adolescents are at high risk of becoming obese in adulthood and more susceptible to the development of CVD. Early cardiovascular disease risk factors identification and IR diagnosis in adolescents is therefore of great value in the prevention of chronic diseases, as it plays a central role in the development of the metabolic disorder [1,46]. In our study, we confirmed a highly significant relationship between IR and overweight/obesity; the mean HOMA-IR value in normal-weight adolescents was only 2.14 ± 1.34, and in adolescents with overweight it was 2.74 ± 1.70, but in adolescents with obesity this value was up to 4.25 ± 3.78. HOMA-IR shows high significant correlations with all anthropometric indicators of overweight/obesity in bivariate analysis. Ashwell et al. [53] consider WHtR as a better screening tool for cardiometabolic risk factor identification compared to waist-to-hip ratio or BMI. It can be used in both adults and children in different ethnic groups. Unlike waist circumference, which increases with age, WHtR almost does not change with age, so it can be considered the best central obesity indicator in adolescents of both sexes. Similar associations have been found by other authors who agree that the waist circumference, and especially WHtR, are good predictors of IR and MetS in adolescents and can be used for simple non-invasive identification of individuals at risk [11,52,54,55]. 

Hyperinsulinemia is an independent risk factor for CVD development because it accelerates the onset of dyslipidemia. Insulin resistance leads to increased fatty acid oxidation, which provides a substrate for the TG synthesis and increases the release of LDL-C into serum [56]. A study evaluating serum insulin levels over eight years in children and young adults showed that dyslipidemias were three times more common in patients with hyperinsulinemia [57]. The bivariate analysis confirmed highly significant associations of IR with dyslipidemia in our cohort, while the multivariate logistic regression confirmed a significant association of IR only with triglyceride levels in both sexes and with non-HDL-C in females. Triglyceride levels remained the only high significant value among plasma lipids even after backward stepwise logistic regression analysis. In a large sample of the Korean population, Kim et al. [58] showed significant positive associations of IR measured as HOMA-IR with waist circumference, BMI, TC, and TG, as well as TG/HDL-C ratio and negative associations with HDL-C, whereas IR was significantly associated with TG/HDL-C ratio, regardless of the waist circumference.

Adolescence is characterized by increased vulnerability and exposure to stress and is considered one of the most difficult periods of life, as many changes take place at several levels (e.g., psychological, physical, environmental, and social). The HELENA study, conducted in a large sample of adolescents aged 12.5–17.5 years from six European cities, found that females experienced more stress than males [59], which is probably related to higher adipose tissue in adolescent girls compared to boys [60]. These findings support the hypothesis that stress may promote excessive fat storage in the body via the interrelationships of the stress system and the mechanisms of energy homeostasis and IR. In our cohort, a significantly higher stress load of females and a significantly higher prevalence of overweight/obesity according to body fat content in females were confirmed. However, paradoxically, a significant inverse association of IR with the experience of frequent stressful situations at school in females was found. 

Sleep is an important factor for normal growth and development during childhood and adolescence [28] and is related to physical, cognitive, emotional, and social development. Sleep depression is considered an independent risk factor for IR development. Several data refer to an association of sleep duration reduction with decreased insulin sensitivity. It is thought that interventions to sleep prolongation could reduce diabetes and obesity risk in adolescents [61]. We found a significant association between shorter sleep duration and HOMA-IR during the weekends only in males; this relationship was not confirmed in females. In contrast, Dorenbos et al. [61], in a small group of 137 children (mean age of 14.4 years), confirmed that sleep duration and quality are important factors that affect insulin sensitivity.

Another lifestyle factor that can directly or indirectly affect insulin sensitivity is physical activity. Several studies have found that increased physical effort increases insulin sensitivity in overweight adolescents, even when there is no change in body weight or body fat content [61]. Sedentary behavior characterized by activities with low energy expenditure (sitting or lying down, watching TV, sitting in class, etc.) is a relatively new risk factor for CVD in later life and attracts considerable attention as a potential risk factor for obesity in young people [62]. We confirmed a highly significant association of IR with insufficient physical fitness and insufficient physical activity in both sexes. In contrast, the sedentary activities duration (learning, watching TV, working/gaming on a PC) did not confirm any association with IR in either sexes, neither separately nor as the sum of all sedentary activity durations. On the other hand, Altenburg et al. [63] found in a sample of young adults aged 18–28 years that cardiometabolic biomarkers significantly positively correlated with TV watching, but not with the time spent at the computer. They therefore recommend that these two variables should be assessed separately. This could be a common impact of TV watching and consumption of high-energy and often-advertised foods; adolescents who declared frequent TV watching declared also a higher consumption of fatty foods, fast foods, and sugary drinks, and less fruit and vegetables [64,65]. Coombs and Stamatakis [62] came to similar conclusions. On the other hand, Fletcher et al. [66] found moderate to strong evidence of a relationship between watching TV, overall sedentary behavior, and obesity, independently of eating habits. 

Smoking is an important risky behavior in adolescents worldwide, with potential adverse health effects. It is assumed that early smoking onset age is an important public health issue, but there are controversial outcomes regarding the health consequences of adolescents [67]. Kelishadi et al. [68] confirmed, in 5625 Iranian students aged 10–18 years, increased risk of MetS and some other cardiometabolic risk factors in smokers (active as well as passive). However, our results are controversial; male smokers (current/former) had less odds of having IR than non-smokers. These inconsistent findings can be explained by relatively short smoking time, a low number of cigarettes smoked, and the definition of a current smoker (any cigarette in the past month). Despite these results, tobacco use prevention among adolescents has to be reinforced.

One of the main causes of obesity, IR, and MetS is generally considered to be related to food choices and eating habits. It is even assumed that there is some connection between sleep deprivation and food choice, i.e., that people with sleep deprivation more often tend to choose unhealthy foods high in energy and fat content [61]. Breakfast is commonly considered to be a key component of a healthy diet that contributes to the all-day diet adequacy and psychological wellbeing [69]. Adolescents who frequently skip breakfasts are thought to be more likely to drink alcohol and smoke and have inadequate physical activity compared to regular breakfast consumers who have shown higher cardiorespiratory fitness. Several studies have also confirmed an association between skipping breakfast and overweight/obesity development in adolescents [70,71]. We confirmed a significant inverse association between regular breakfast consumption and IR in males, as well as a significant inverse association between IR and the total number of meals per day and a significant positive association with frequent sweetened beverage consumption in females. A backward stepwise logistic regression analysis in the whole sample of adolescents confirmed the significance of skipping breakfast and frequent sweetened beverages consumption (daily/several times a week) in IR development. A significant relationship between the frequency of sweetened beverages consumption and HOMA-IR was also confirmed in the HELENA study [72]. It is well known that excess sugar intake has harmful effects on CVD risk factors and that there is a strong link between added sugar consumption with MetS [73]. We have not identified significant associations of IR with any other food commodities, probably due to skewed food consumption data (adolescents with overweight/obesity underestimated consumption of obesogenic food groups), which is typical behavior in individuals with overweight/obesity [74]. 

Family history, ethnicity, prenatal and postnatal nutrition, obesity, pubescence, eating habits, and sedentary lifestyle may affect insulin sensitivity in the pediatric population [45]. It is argued that low birth weight may affect the risk of CVD and that breastfeeding may protect against the development of type 2 diabetes, CVD, and obesity [75]. Of these perinatal factors, we can confirm (but only in bivariate analysis) a significant association of HOMA-IR with the low birth weight of boys. We did not find any significant differences concerning breastfeeding duration. Similarly, no effect of breastfeeding on IR was confirmed by Huybrechts et al. [60] in the HELENA study. We also monitored the associations of IR with the current weight and educational level of both parents from the family questionnaire. A study of more than 1000 Canadian children and adolescents showed that families with a higher level of education had a lower incidence of one or more MetS risk factors compared to families with a lower educational level. This can be attributed to the impact of higher educational level on health awareness and habits such as eating habits and physical activity [76]. It is therefore recommended that low-educated parents raise awareness of early CVD risk in their children [77]. A significant relationship was observed between parental education and the weight of children in a group of more than 1500 Sicilian children—the obesity prevalence was significantly higher regarding low education of the mother or father [78]. According to these authors, the results suggest that having a mother with a lower level of education can be considered a risk factor for childhood obesity. However, in a previous study in northern Tuscany, the authors found that the impact of a father’s education is stronger than that of the mother [79]. We can confirm these results; in our study a significant inverse association between the higher education of fathers and IR prevalence was found. This relationship was the opposite concerning the mother’s education. A significant relationship between IR and the higher weight of both parents was found only in males. 

The strength of our study is in a large sample of adolescents and young adults and the ability to examine a wide variety of cardiometabolic and selected lifestyle risk factors, as well as selected factors of personal and family history in males and females. These indicators are of great importance for CVD prevention in adolescents and young adults. Our study is limited by its cross-sectional design, which does not determine cause-and-effect relationships between different variables. This method is often used to make inferences about possible relationships or to gather preliminary data to support further research and experimentation. The generalizability of our findings to the wider population may be limited as well. The other limitations are mentioned in the discussion. 

## 5. Conclusions

In our study, a relatively high incidence of IR (in a total of 18.6% of adolescents) without significant sex differences was found in a large sample of adolescents in the Slovakian capital self-governing region. In both sexes, the highly significant associations of IR with overweight/obesity, specifically with abdominal obesity expressed as WHtR, were confirmed. Regarding plasma lipids, triglyceride levels had the closest association with IR. When it comes to lifestyle factors, a highly significant relationship of IR with insufficient physical activity and low physical fitness in both sexes was found, but we were unable to confirm the impact of daily sedentary activity duration. Skipping breakfast, a low number of meals per day (only in females), and frequent consumption of sweetened beverages showed significant relationships with IR regarding dietary habits. Our study also found an important association of parents’ body weight and parental educational level (especially of fathers) with the development of IR in adolescents. On the other hand, we cannot confirm the impact of low birth weight and/or breastfeeding duration on IR in adolescents.

## Figures and Tables

**Table 1:** Basic sample characteristics and sex differences (n = 2629).

Variable		Males	Females	*p*_1_
n		1205	1424	-
Age	(years)	17.1 ± 1.0	17.1 ± 1.0	0.289
Body mass index	(kg.m^−2^)	22.31(20.51, 24.75)	21.30 (19.65, 23.66)	<0.001
Z-score BMI		0.37 (−0.22, 1.16)	0.16 (−0.38, 0.91)	<0.001
Total body fat	(%)	17.6 ± 7.4	30.4 ± 6.9	<0.001
Waist circumference	(cm)	79.3 ± 9.2	71.5 ± 7.8	<0.001
Waist/height		0.44 ± 0.05	0.43 ± 0.05	<0.001
Cholesterol, total	(mmol/L)	3.80 ± 0.69	4.24 ± 0.75	<0.001
LDL-cholesterol	(mmol/L)	2.16 ± 0.58	2.34 ± 0.60	<0.001
HDL-cholesterol	(mmol/L)	1.25 ± 0.23	1.50 ± 0.30	<0.001
Non-HDL-cholesterol	(mmol/L)	2.55 ± 0.67	2.73 ± 0.68	<0.001
Triglycerides	(mmol/L)	0.75 (0.58, 1.01)	0.79 (0.60, 1.04)	0.036
Atherogenic index		−0.20 ± 0.23	−0.26 ± 0.20	<0.001
Glucose	(mmol/L)	4.93 ± 0.44	4.71 ± 0.75	<0.001
hsCRP	(mg/L)	0.43 (0.19–1.15)	0.46 (0.20, 1.19)	0.168
Insulin	(mIU/L)	9.28 (6.87, 12.65)	9.86 (7.41, 13.41)	0.002
HOMA-IR		2.53 ± 2.25	2.39 ± 1.58	0.077
Systolic BP	(mmHg)	122.6 ± 12.1	107.3 ± 9.4	<0.001
Diastolic BP	(mmHg)	72.7 ± 7.9	70.4 ± 7.6	<0.001
Heart rate	(min^−1^)	78.0 ± 13.1	81.1 ± 12.4	<0.001
Ruffier index		9.2 ± 4.0	10.5 ± 4.1	<0.001
**High-risk markers prevalence**				***p*_2_**
Body mass index	overweight; n (%)	212 (17.6)	214 (15.0)	0.075
	obesity; n (%)	158 (13.1)	112 (7.9)	<0.001
Body fat content	overweight; n (%)	94 (7.9)	195 (14.0)	<0.001
	obesity; n (%)	144 (12.2)	201 (14.4)	0.099
Waist	central obesity; n (%)	115 (9.5)	181 (12.7)	0.085
Waist/height	≥0.5; n (%)	148 (12.3)	119 (8.4)	<0.001
Total cholesterol	≥4.85 mmol/L; n (%)	92 (7.6)	272 (19.1)	<0.001
LDL-cholesterol	≥3.25 mmol/L; n (%)	51 (4.2)	100 (7.0)	0.002
HDL-cholesterol	≤0.85 mmol/L; n (%)	36 (3.0)	7 (0.5)	<0.001
Non-HDL-cholesterol	>3.70 mmol/L; n (%)	67 (5.6)	114 (8.0)	0.014
Triglycerides	≥1.50 mmol/L; n (%)	81 (6.7)	107 (7.5)	0.432
Atherogenic index	≥0.21; n (%)	53 (4.4)	23 (1.6)	<0.001
Glucose	≥5.6 mmol/L; n (%)	55 (4.6)	25 (1.8)	<0.001
hsCRP	>3 mg/L; n (%)	92 (7.6)	153 (10.7)	0.007
Insulin	≥20 mIU/L; n (%)	100 (8.3)	89 (6.2)	0.036
HOMA-IR	≥3.16; n (%)	238 (19.8)	251 (17.6)	0.147
Systolic BP	hypertension range; n (%)	147 (12.2)	17 (1.2)	<0.001
Diastolic BP	hypertension range; n (%)	53 (4.4)	50 (3.5)	0.242
Physical fitness	weak/insufficient n (%)	461 (39.2)	710 (52.6)	<0.001
Metabolic syndrome	IDF consensus; n (%)	32 (2.7)	6 (0.4)	<0.001

BMI—body mass index; LDL—low-density lipoprotein; HDL—high-density lipoprotein; hsCRP—high-sensitivity C-reactive protein; HOMA—Homeostasis Model Assessment; IR—insulin resistance; BP—blood pressure; IDF—International Diabetes Federation; *p*_1_—two-sample *t*-test; *p*_2_—chi-square test. Data are presented as mean ± standard deviation for normal distribution, as median (lower quartile–upper quartile) for non-normal distribution, or as count (percentage) for categorical data.

**Table 2:** Selected lifestyle characteristics and eating habits in males and females (n = 2629).

Variable		Males	Females	*p*
n		1.205	1.424	
Smokers ^1^ (current/former)	n (%)	433 (35.9)	569 (40,0)	0.031
Physical activity (duration per week)	(min)	300.0 (90.0, 45.0)	78.0 (16.1, 218.0)	<0.001
Sleeping duration (Mon-Fri)	(h)	7.2 ± 1.1	7.1 ± 1.1	0.001
Sleeping duration (weekends)	(h)	9.1 ± 1.4	9.3 ± 1.4	0.002
Working/gaming at the computer (Mon-Fri)	(h)	3.4 ± 1.9	3.0 ± 1.8	<0.001
Working/gaming at the computer (weekends)	(h)	4.6 ± 2.6	3.8 ± 2.3	<0.001
Watching TV (Mon-Fri)	(h)	1.9 ± 1.6	2.0 ± 1.6	0.133
Watching TV (weekends)	(h)	2.8 ± 2.2	3.0 ± 2.0	0.002
Learning (Mon-Fri)	(h)	1.1 ± 0.9	1.8 ± 1.1	<0.001
Learning (weekends)	(h)	1.1 ± 1.0	1.8 ± 1.2	<0.001
Sedentary activities overall (Mon-Fri)	(h)	6.2 ± 2.7	6.7 ± 3.0	<0.001
Sedentary activities overall (weekends)	(h)	8.3 ± 3.7	8.5 ± 3.7	0.114
Frequent stress situations at school	n (%)	243 (20.7)	436 (31.0)	<0.001
Frequent stress situations in privacy	n (%)	79 (6.9)	127 (9.2)	0.030
The average number of meals per day		4.1 ± 1.4	4.0 ± 1.3	0.004
Breakfast consumption (regularly, daily)	n (%)	561 (52.7)	554 (43.7)	<0.001

Mon–Fri—working days; TV—television. Data are presented as mean ± standard deviation for normal distribution, as median (lower quartile–upper quartile) for non-normal distribution, or as count (percentage) for categorical data. ^1^ smoker—any cigarette during the past month.

**Table 3:** Selected cardiometabolic variables (normal/acceptable vs. borderline/high risky) associated with insulin resistance (defined according to HOMA-IR) in males (n = 1205) and females (n = 1424)—multivariate logistic regression analysis.

Selected Cardiometabolic Variables		AOR	95% CI	AOR	95% CI
		Males		Females	
Total cholesterol	<4.10 mmol/L	1		1	
	≥4.10 mmol/L	0.87	0.53, 1.44	0.71	0.49, 1.04
HDL-cholesterol	>1.10 mmol/L m. >1.25 mmol/L f.	1	-	1	-
	≤1.10 mmol/L m. ≤1.25 mmol/L f.	1.32	0.91, 1.91	1.07	0.74, 1.55
nonHDL-cholesterol	<3.2 mmol/L	1	-	1	-
	≥3.2 mmol/L	0.93	0.51, 1.69	1.50	1.00, 2.26 *
Triglycerides	<1.15 mmol/L	1	-	1	-
	≥1.15 mmol/L	3.77	2.28, 6.22 ***	1.60	1.08, 2.38 *
Atherogenic index AIP	<0.11	1	-	1	-
	≥0.11	0.93	0.48, 1.78	1.71	0.92, 3.18
High-sensitive C-reactive protein	<1mg/L	1	-	1	-
	≥1mg/L	1.23	0.85, 1.78	1.20	0.87, 1.66
Systolic blood pressure	<90. percentile	1	-	1	-
	≥90. percentile	1.40	0.97, 2.04	1.34	0.63, 2.85
Body fat content (%)	normal/underweight	1	-	1	-
	overweight/obesity	2.64	1.66, 4.21 ***	2.29	1.65, 3.19 ***
Waist/height ratio	≤0.5	1	-	1	-
	>0.5	3.25	1.91, 5.51 ***	1.96	1.24, 3.11 **

* *p* < 0.05; ** *p* < 0.01; *** *p* < 0.001; AOR—adjusted odds ratio for all variables in the model; CI—confidence interval; HDL—high-density lipoprotein; m.—males; f.—females.

**Table 4:** Selected lifestyle variables (acceptable vs. risky) associated with insulin resistance (defined according to HOMA-IR) in males (n = 1205) and females (n = 1424)—multivariate logistic regression analysis.

Selected Lifestyle Variables		AOR	95% CI	AOR	95% CI
		Males		Females	
Ruffier index	≤10	1	-	1	-
	>10	2.13	1.49, 3.04 ***	1.45	1.04,2.02 *
Physical activity duration/week	≥225 min	1	-	1	-
	<225 min	1.81	1.27, 2.57 **	1.75	1.13,2.72 *
Number of meals/day	≥3-4	1	-	1	-
	<3-4	0.89	0.45, 1.76	2.45	1.47,4.08 **
Breakfast consumption	daily	1	-	1	-
	occasionally/not at all	1.54	1.08, 2.18 *	1.13	0.80,1.60
Sweetened beverages consumption	exceptionally/not at all	1	-	1	-
	daily/several times a week	0.87	0.58, 1.30	1.54	1.08,2.18 *
Smoking (current/former)	no	1	-	1	-
	yes	0.66	0.45, 0.97 *	0.78	0.54,1.10
Frequent stress situations at school	no/exceptionally	1	-	1	-
	sometimes/often	1.00	0.68, 1.47	0.59	0.39,0.87 **
Frequent stress situations in privacy	no/exceptionally	1	-	1	-
	sometimes/often	1.22	0.84, 1.78	1.04	0.74,1.45
Sleeping duration (Mon-Fri)	≥8 h	1	-	1	-
	<8 h	1.41	0.97, 2.04	0.93	0.66,1.31
Sleeping duration (weekends)	≥8 h	1	-	1	-
	<8 h	1.75	1.01, 3.01 *	0.96	0.51,1.80
Learning duration (Mon-Fri)	≤2 h	1	-	1	-
	>2 h	0.79	0.33, 1.87	1.02	0.64,1.61
Learning duration (weekends)	≤2 h	1	-	1	-
	>2 h	1.00	0.50, 2.00	0.69	0.44,1.08
Working/gaming at the computer (Mon-Fri)	≤2 h	1	-	1	-
	>2 h	0.94	0.62, 1.43	0.86	0.60,1.24
Working/gaming at the computer (weekends)	≤2 h	1	-	1	-
	>2 h	1.27	0.76, 2.12	1.11	0.75,1.64
Watching TV (Mon-Fri)	≤2 h	1	-	1	-
	>2 h	1.25	0.78, 2.01	0.90	0.60,1.42
Watching TV (weekends)	≤2 h	1	-	1	-
	>2 h	1.06	0.70, 1.60	1.36	0.94,1.97

* *p* < 0.05; ** *p* < 0.01; *** *p* < 0.001; AOR—adjusted odds ratio for all variables in the model; CI—confidence interval; Mon—Monday; Fri—Friday.

**Table 5:** Selected variables of adolescents’ personal and family history associated with insulin resistance (defined according to HOMA-IR) in males (n = 1205) and females (n = 1424)—multivariate logistic regression analysis.

Selected Factors of Personal and Family History		AOR	95% CI	AOR	95% CI
		Males		Females	
Birth weight	>2500g	1	-	1	-
	≤2500g	1.32	0.58, 3.01	1.18	0.61, 2.27
Breastfeeding duration	>3 months	1	-	1	-
	≤3 months	1.15	0.83, 1.59	0.98	0.72, 1.33
Father’s educational level ^†^	university/higher vocational	1	-	1	-
	basic/secondary	1.35	0.94, 1.94	1.84	1.24, 2.73 **
Mother’s educational level ^†^	university/higher vocational	1	-	1	-
	basic/secondary	0.62	0.44, 0.88 **	0.97	0.68, 1.37
Father’s current weight ^†^	≤90kg	1	-	1	-
	>90kg	1.39	1.01, 1.90 *	1.06	0.78, 1.44
Mother’s current weight ^†^	≤70kg	1	-	1	-
	>70kg	1.79	1.30, 2.46 ***	1.15	0.84, 1.57

* *p* < 0.05; ** *p* < 0.01; *** *p* < 0.001; AOR—adjusted odds ratio for all variables in the model; CI—confidence interval; ^†^ There are some missing data in this variable category (adolescent has no father and/or mother).

**Table 6:** Variables significantly associated with insulin resistance (defined according to HOMA-IR) in adolescents (n = 2629)—backward stepwise logistic regression analysis, age, and sex-adjusted.

Variables		AOR	95% CI
Sex	females	1	-
	males	1.35	0.99, 1.83 ^1^
Age group	<17 years	1	-
	≥17 years	0.70	0.53, 0.92 *
HDL-cholesterol	>1.10 mmol/L m. >1.25 mmol/L f.	1	-
	≤1.10 mmol/L m. ≤1.25 mmol/L f.	1.36	0.99, 1.86 ^2^
Triglycerides	<1.15 mmol/L	1	-
	≥1.15 mmol/L	2.77	2.02, 3.80 ***
Body fat content (%)	normal/underweight	1	-
	overweight/obesity	2.45	1.75, 3.43 ***
Waist/height ratio	≤0.5	1	-
	>0.5	3.20	2.08, 4.94 ***
Ruffier index	≤10	1	-
	>10	1.55	1.17, 2.06 **
Physical activity duration/week	≥225 min	1	-
	<225 min	1.79	1.29, 2.48 ***
Breakfast consumption	daily	1	-
	occasionally/not at all	1.42	1.08, 1.88 *
Sweetened beverages consumption	exceptionally/not at all	1	-
	daily/several times a week	1.50	1.11, 2.08 **
Smoking (current/former)	no	1	-
	yes	0.71	0.53, 0.95 *
Frequent stress situations at school	no/exceptionally	1	-
	sometimes/often	0.70	0.51, 0.96 *
Father’s educational level ^†^	university/higher vocational	1	-
	basic/secondary	1.46	1.05, 2.03 *
Mother’s educational level ^†^	university/higher vocational	1	-
	basic/secondary	0.72	0.53, 0.98 *

* *p* < 0.05; ** *p* < 0.01; *** *p* < 0.001; ^1^ *p* = 0.054; ^2^ *p* = 0.053; AOR—adjusted odds ratio; CI—confidence interval; HDL—high-density lipoprotein; m.—males; f.—females; ^†^ there are some missing data in this variable category (adolescent has no father and/or no mother).

## Data Availability

The data presented in this study are available on request from the corresponding author.
